# Vasopressin as Possible Treatment Option in Autism Spectrum Disorder

**DOI:** 10.3390/biomedicines11102603

**Published:** 2023-09-22

**Authors:** Kristóf László, Dávid Vörös, Pedro Correia, Csilla Lea Fazekas, Bibiána Török, Imola Plangár, Dóra Zelena

**Affiliations:** 1 Institute of Physiology, Medical School, University of Pécs, 7624 Pecs, Hungary; kristof.laszlo@aok.pte.hu (K.L.); voros.david@pte.hu (D.V.); correiaufpe@gmail.com (P.C.); fazekas.csilla@pte.hu (C.L.F.); torok.bibiana@koki.hu (B.T.); plangar.imola@pte.hu (I.P.); 2 Center of Neuroscience, University of Pécs, 7624 Pecs, Hungary; 3 Szentágothai Research Center, University of Pécs, 7624 Pecs, Hungary; 4 Hungarian Research Network, Institute of Experimental Medicine, 1083 Budapest, Hungary

**Keywords:** autism spectrum disorder, vasopressin, social behavior, stereotype behavior, medial preoptic area, lateral septum, amygdala

## Abstract

Autism spectrum disorder (ASD) is rather common, presenting with prevalent early problems in social communication and accompanied by repetitive behavior. As vasopressin was implicated not only in salt-water homeostasis and stress-axis regulation, but also in social behavior, its role in the development of ASD might be suggested. In this review, we summarized a wide range of problems associated with ASD to which vasopressin might contribute, from social skills to communication, motor function problems, autonomous nervous system alterations as well as sleep disturbances, and altered sensory information processing. Beside functional connections between vasopressin and ASD, we draw attention to the anatomical background, highlighting several brain areas, including the paraventricular nucleus of the hypothalamus, medial preoptic area, lateral septum, bed nucleus of stria terminalis, amygdala, hippocampus, olfactory bulb and even the cerebellum, either producing vasopressin or containing vasopressinergic receptors (presumably V_1a_). Sex differences in the vasopressinergic system might underline the male prevalence of ASD. Moreover, vasopressin might contribute to the effectiveness of available off-label therapies as well as serve as a possible target for intervention. In this sense, vasopressin, but paradoxically also V_1a_ receptor antagonist, were found to be effective in some clinical trials. We concluded that although vasopressin might be an effective candidate for ASD treatment, we might assume that only a subgroup (e.g., with stress-axis disturbances), a certain sex (most probably males) and a certain brain area (targeting by means of virus vectors) would benefit from this therapy.

## 1. Introduction

The prevalence of autism spectrum disorder (ASD) is unfortunately rather high and increasing [1], with a 4.3:1 boy-to-girl ratio [2] (Figure S1a). Different countries may report rather different data, from 0.02% in China to 3.66% in Sweden [3]. Some publications suggest that the main reason for the uptrend is the change in diagnostic criteria and the rise in awareness [4,5]. However, the role of other factors in this increase cannot be ruled out [6]. For example, both the maternal and paternal age—which is constantly increasing [7]—and the higher socioeconomic status can increase the risk for ASD [8]. The total lifetime estimated cost of ASD increased almost four times over the last 20 years [1] (Figure S1b,d). Accordingly, the interest in ASD is also constantly increasing, with an almost exponential growth in the number of publications from 2000 (Figure S1c).

**Figure 1:**
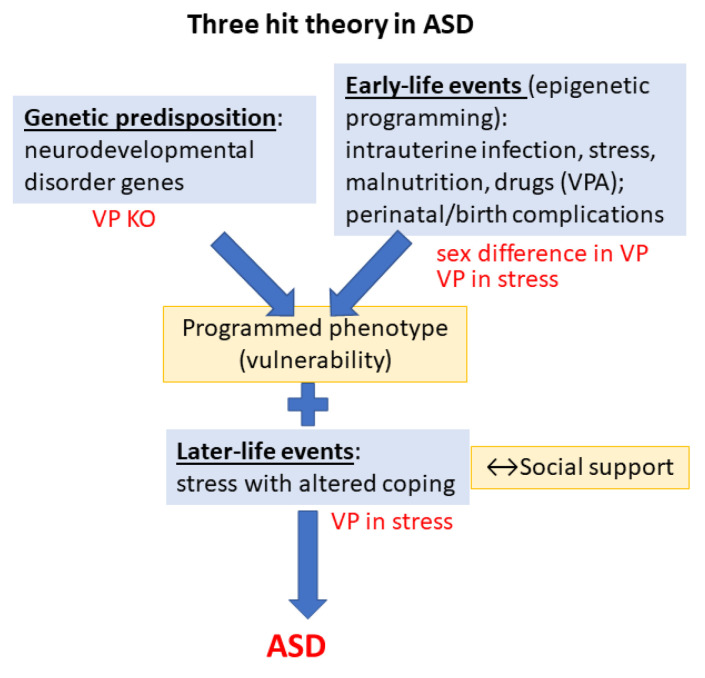
The three-hit theory of autism spectrum disorder. Vasopressin may contribute to the development of symptoms at all levels. Abbreviations: ASD: autism spectrum disorder; VP: vasopressin; KO: knockout; VPA: valproate.

Despite intensive ongoing research, the cause of ASD is still unknown. As most disorders, ASD might also have multiple causes, indicated by the *three-hit theory* [9] (Figure 1). As the *first “hit”*, ASD is associated with many genes, called high-confidence neurodevelopmental disorder genes [10]. From the more than 1500 genes participating in neurodevelopment, 1452 genes are located on the autosomes, 129 on the X chromosome, and 5 on the mitochondrial genome. These genes encode key regulators of synaptogenesis, synaptic plasticity, cytoskeleton dynamics, protein synthesis and degradation, chromatin remodeling, transcription, and lipid homeostasis [11]. The higher prevalence of ASD in boys might be due to X chromosome implication. Indeed, two mutations (neuroligin 3 and 4 (NLGN3 and NLGN4)) of the X chromosome may predispose males to ASD [12]. Interestingly, the number of X chromosomes influenced social behavior with parallel changes in the vasopressin (VP) content of amygdala in mice and VP plasma levels in patients [13]. Moreover, twin studies also supported strong genetic effects in ASD [14]. Interestingly, a recent study found common genetic variants in angiotensin II receptor type 2 in the maternal and infant DNA samples associated with risk of ASD, presumably through its involvement in the maturation of the VP–oxytocin (OT) pathway [15]. Additionally, endocrine (among others VP) and environmental factors might also be responsible for the sex difference (see later) [16].

As the *second “hit”*, numerous early developmental risk factors were identified. A meta-analysis pointed out the fetus’s abnormal presentation, umbilical cord complications, fetal distress, birth injury or trauma, multiple births, maternal hemorrhage, summer birth, low birth weight, small for gestational age, congenital malformation, low 5 min Apgar score, feeding difficulties, meconium aspiration, neonatal anemia, ABO or Rh incompatibility, and hyperbilirubinemia as possible harmful perinatal events [17]. However, earlier intrauterine events might be similarly important, such as infections [18], and other maternal immune dysregulations [19], prenatal stress [19], malnutrition [20] or different drugs, like the antiepileptic valproate (VPA) [21]. The administration of VPA, a known histone deacetylase inhibitor, into the 11.5–13.5-day pregnant rodent mother influences the epigenetic machinery and induces ASD-like changes in the offspring, being a widely used animal model of ASD [22,23]. Interestingly, when acute VPA injection was used as gamma-aminobutyric acid (GABA) agonist, it was able to diminish angiotensin II- as well as hyperosmotic stimulus-induced VP rise in normal men [24,25]. 

As for the *third “hit”* (exacerbating acute stress), the measles, mumps, and rubella (MMR) vaccine was accused by the Wakefield report to increase the chance of developing ASD [26]. However, further studies contradicted this hypothesis [27,28]. Nevertheless, ASD patients may be more vulnerable to stress, with higher prevalence of, e.g., post-traumatic stress disorder [29]. In support, their hypothalamic–pituitary–adrenal (HPA) axis and autonomic nervous system (ANS) exhibit atypical functions both at resting state and during the presence of social and/or non-social stressors [30]. Higher levels of perceived stress and difficulties with coping have been reported in ASD children and adults [31].

It is more of a rule than an exception that people with ASD have at least one comorbid psychiatric disorder (80.9% of patient), with anxiety disorder being especially frequent (55.3%) [32,33,34]. Along the comorbidities, the prevalence of epilepsy [35], anxiety disorder [32], depression, obsessive compulsive disorder (OCD), specific phobias, and attention deficit hyperactivity disorder (ADHD) is higher than in the normal population [36,37,38]. For example, 33–37% of children with ASD present ADHD symptoms [39]. This makes the pathomechanism and therapies even more difficult.

Our aim was to summarize the literature exploring the possible symptoms of ASD and frequent comorbidities with a known or suggested contribution of VP and the possible therapeutic considerations. Previous studies focused either on the role of OT [40], on social skills [41] or on treatment options [42], without attempting to present a comprehensive view on VP–ASD interaction. 

## 2. Major Symptoms of Autism Spectrum Disorder

**Figure 2:**
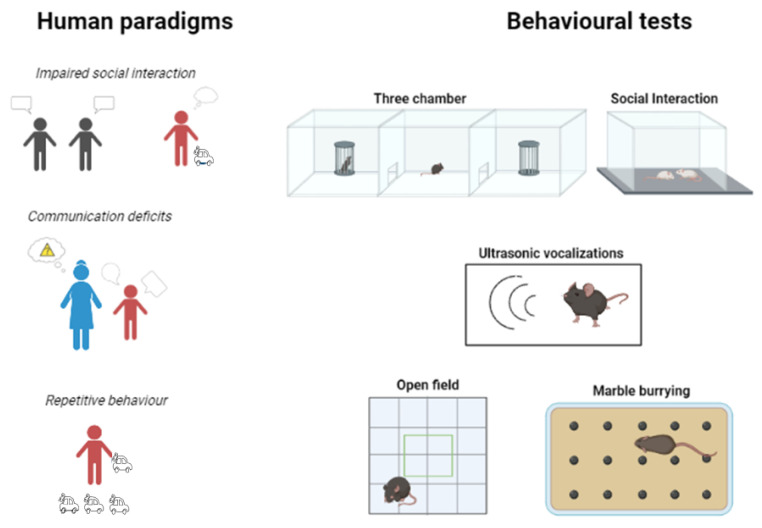
Comparison of core human symptoms with animal tests modeling them. Impaired social interaction can be modeled via the three-chamber sociability test or anxiogenic social interaction in a new cage. Communication can be measured using ultrasound vocalization, while enhanced allo-grooming in rodents—observable in an open-field—is a sign of stereotyped, repetitive behavior together with the increased number of buried marbles during the marble burying test.

According to the Diagnostic and Statistical Manual of Mental Disorders Fifth Edition Text Revision (DMS-5-TR) released in 2022, ASD is a pervasive neurodevelopmental disorder (NDD). Differences in behavior and developmental milestones appear in affected children by the first year of life [43,44]. The core symptoms are (1) deficits in social communication (e.g., disrupted language skills) together with anomalous socioemotional responses in several contexts; and (2) restricted, repetitive patterns of behavior [45,46] (Figure 2). Inaccurate parental recall can hinder the diagnosis [47,48]. The transition to prospective studies of high-risk infants has enabled a more comprehensive identification of early characteristics. 

**Figure 3:**
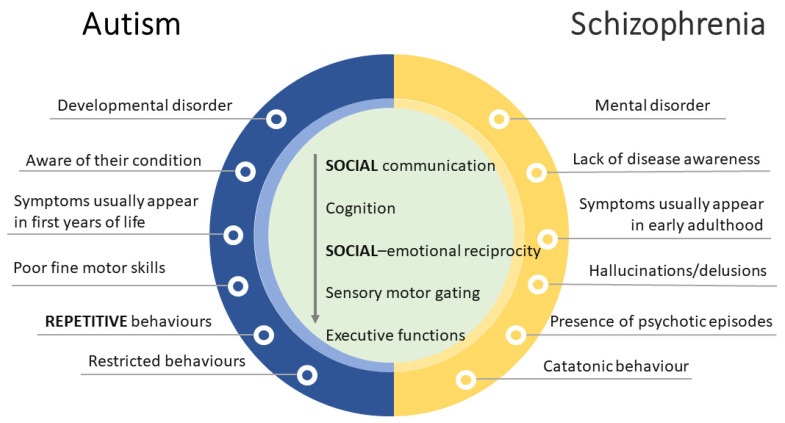
Comparison of autism spectrum disorder (ASD) and schizophrenia (SCZ). Despite core similarities in social and cognitive domains, several unique features can be observed making the differential diagnosis easier.

The large variation in the severity of symptoms within and across different groups hampered the ability to distinguish one disorder from another [49]. Of note, ASD and schizophrenia (SCZ) seem to overlap on many levels. Indeed, before the publication of the DSM-III [50], when ASD was first introduced as a different clinical diagnosis, autistic children were frequently diagnosed with childhood SCZ, which is characterized by abnormal perceptions of reality as well as deficits in social functioning [51]. Though ASD and SCZ are now classified as distinct disorders, they frequently co-occur and share common genetic risk factors and symptom presentations [52] (Figure 3).

In 1994, the categorical diagnoses of Asperger’s disorder, childhood disintegrative disorder, Rett’s disorder, and Pervasive Developmental Disorder not otherwise specified were introduced [53]. These disorders are distinguished by three core deficits: (1) impaired reciprocal social interaction, (2) deficient communication, and (3) restricted/repetitive behavioral or interest patterns [54] (Table 1, Figure 2). The range and severity of these impairments vary, and they frequently change with the acquisition of other developmental skills [55].

The 5th edition of the DSM altered the diagnostic criteria and put all the previous mentioned disorders (excluding SCZ) under the ASD category. The number of deficits in the core domains has been reduced to two ((1) social communication and (2) repetitive behavior; see earlier). ASD was diagnosed if a patient showed at least three social communication symptoms (e.g., reduced eye contact, lack of facial expressions, and impaired ability to start or maintain a conversation with others) and at least two restricted interests/repetitive behaviors, with an added behavior of hyper- or hypo-reactivity to sensory input or unique interests in sensory parts of the environment [56]. In the latest edition, the DSM-5-TR, some wordings were changed (e.g., *“all of the following”* criteria must be fulfilled) to tighten the diagnosis, thereby avoiding overdiagnosis.

### Symptoms in Animal Models

To better understand the mechanism and identify new treatment options, animal models are needed. ASD models can be divided into two categories: genetical (e.g., mutation in OTR [57], NLGN, SRC homology 3 and multiple ankyrin repeat domains protein (SHANK), Contactin Associated Protein 2 (CNTNAP2), Melanoma Antigen Gene Family Member L2 (MAGEL2), a zinc-finger transcription factor TSHZ3, fragile-X syndrome [58,59]) and environmental [22]. The later can be drug-induced (e.g., VPA), immunological (polyriboinosinic: polyribocytidylic acid (poly I:C), a kind of maternal immune activation (MIA) [60]; at embryonic age E 11.5–13.5) or developmental, lesion-induced (presumably hippocampus lesion [61], the CA2 region [62], where V_1b_ receptors might regulate aggression [63,64]). 

A wide range of species is used. The most frequent are rodents (presumable transgenic mice [58] and rats), but avian [65], *Drosophila melanogaster* [11] and even zebrafish models [66,67] are also available [59].

Researchers can detect some of the behavioral components of ASD using a range of behavioral tasks in animal models (Table 1) [68]. Sociability, repetitive behavior, narrowness of the interest and associated symptoms (e.g., anxiety) are some of the parameters that are widely studied [69]. For example, the three-chamber sociability test [70] measures overall sociability and interest through direct social approach behaviors when a subject is given the option of spending time with a stimulus animal or an object. Measuring ultrasonic vocalizations [71] and grooming during an open-field test (OFT) [72] are two examples of behavioral tasks with face validity used to study communication deficits and repetitive behavior as well as comorbid anxiety in ASD animal models.

**Table 1:** Comparison between human symptoms and animal models.

Symptoms in Humans		Behavioral Test	Main Parameters in Rodents	References
Impaired social interaction		Three-chamber sociability test	Time and fr. near stimulus animal	[70]
		Social interaction test	Time and fr. of social interactions	
Deficient communication		MS-USV	USV	[71]
Repetitive behavior		MBT	Number of buried marbles, time of digging	[73]
		Self-grooming	Grooming time and fr. in OFT	[74]
Comorbidity	Motor functions	Rotarod	Latency to fall	[75]
		Erasmus Ladder	Number of missteps	[76]
		DigiGait	Stance stride length and steps/sec	[77]
		Delay eyeblink conditioning test	Successful conditioned response, blink amplitude and blinking speed	[77]
	Anxiety	OFT	Total distance, time in center	[72]
		EPM	Time and fr. in arms	[72]
	Pain sensitivity	Hot plate test	Withdraw latency	[78]
		Tail flick test	Withdraw latency	[78]

Abbreviations: EPM: elevated plus maze; fr.: frequency; MBT: marble burying test; MS-USV: maternal separation-induced ultrasonic vocalization; OFT: open-field test; USV: ultrasonic vocalization.

## 3. Vasopressin

Our behavior is regulated by neuronal communication by means of more than 100 identified neurotransmitters [79]. While only 12 classical, small molecule neurotransmitters have been identified, there is an arsenal of oligopeptides that are also utilized by neurons to convey information. One of the best known pairs is the VP-OT, which are famous for their extensive role in physiology. These two neurotransmitters have highly similar amino acid sequences. Their characteristic is a disulfide bridge between position 1 and 6, thereby forming a ring. In mammals, VP and OT differ in two amino acids only: on position 3 (Phe for VP, Ile for OT) and 8 (Arg for VP, Leu for OT). Interestingly, this sequence seems to be exceptionally conserved throughout the species and even along different phylums. There is evidence of so-called VP- or OT-like peptides in invertebrates [80,81] going back on the phylogenetic tree as far as the Hydras of the Cnidarias [82]. The differences between them are also rather subtle, and all variants hold similar molecular properties (such as charge, hydrophobicity, polarity, etc.) [80]. According to our current knowledge, the common ancestral gene (along with their receptors [83]) went through duplication, and, over the course of the vertebrate evolution, the VP and OT genes gained their tail-to-tail orientation [84,85,86]. 

### 3.1. Vasopressin Receptors

When released from the neurohypophyseal nerve terminals of the magnocellular neurons of the paraventricular (PVN) and supraoptic nucleus (SON) of the hypothalamus to the blood, the main effect of the VP is osmoregulation in the kidney [87,88,89]. The activation of *V*_2_ *receptors* (G_s_ pathway) facilitates water reabsorption by increasing the expression and insertion of aquaporin 2 channel into the apical membranes of the collecting duct. Moreover, it also regulates the transcription of urea transporters and sodium channels, resulting in an overall increased water uptake from the urine (thus, the other name of VP is antidiuretic hormone, ADH).

There are two other known receptors of VP that activate the G_q_ (phosphatidylinositol) pathway. *V*_1*a*_ *receptors* can be found in the vessels, liver and brain, with a widespread role in vasoconstriction, gluconeogenesis, blood clotting, social recognition and circadian rhythmicity, among others. On the other hand, *V*_1*b*_ *(or V_3_) receptors* are mainly (but not exclusively) found on the anterior lobe of the pituitary, playing a role in stress adaptation. 

Due to their shared structure, VP and OT can bind to each other’s receptors; however, this requires higher doses. The *OT receptor* (OTR) is also a G-protein coupled receptor [90]. Interestingly, its signalization may switch from G_q_ to G_i_ during pregnancy [91].

### 3.2. Vasopressin and Autism

**Figure 4:**
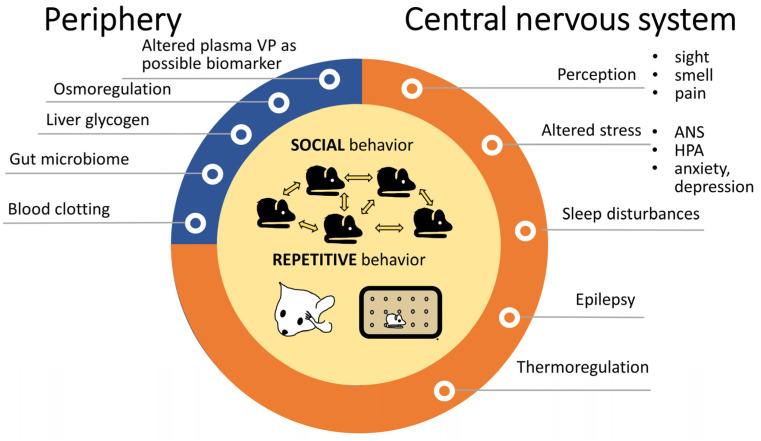
Alterations in autism spectrum disorders with possible contribution of vasopressin. The observations were mainly in animals. Both social problems and repetitive behaviors—depicted in the middle—are core features of autism spectrum disorders and VP is obviously implicated in them. Peripheral VP functions (blue) might be only indirectly linked to autism, while other central VP effects (orange) might have a more important, although not yet fully clarified role. Abbreviations: ANS: autonomous nervous system; HPA: hypothalamic pituitary adrenocortical axis, VP: vasopressin.

Since 2006, a yearly average of 15.44 ± 1.34 articles are published on the possible contribution of VP in ASD (PubMed search with keywords “autism” and “vasopressin” and “publication date”), which shows a constant, stable interest. Vasopressin may influence ASD symptoms at several points, and is not restricted to the two major domains, the social skills and repetitive movements (see Section 2) (Figure 4).

#### 3.2.1. Peripheral Vasopressin Function and Autism

In humans, the classical function of VP is the regulation of the salt-water homeostasis at the periphery (see earlier) and plasma levels are easier to monitor than brain levels. Therefore, human research initially focused on the correlation between peripheral VP concentration and ASD symptoms. 

The VP-ASD connection was first raised in the abstract of a 1992 deWied article without further explanation [92]. In the same year, a brief report provided the first evidence for elevated VP plasma levels in autistic children (one girl and three boys, 11.75 ± 3.06 years old); however, it seemed to be a consequence of the disorder rather than a cause [93]. Moreover, in a later study, only 50% (out of 10) children had higher VP levels, while 70% showed a decrease in adrenocorticotropin (ACTH) levels, the hypophyseal component of the HPA axis [94], suggesting a blunted central VP effect. In some human cases, the blood VP levels correlated with the severity of certain aspects of ASD [95,96]. Maternal levels of VP were also associated with the symptoms of the offspring, with lower levels in the mother of ASD children [96,97]. Moreover, in human peripheral blood mononuclear cells, the expression of V_1a_ receptor positively correlated with better social and behavioral function in ASD children (3–16-year-old) [98]. Despite this evidence, little is known about how altered peripheral VP homeostasis might influence the behavior in ASD patients. 

A possible connection is through osmoregulation, although, for now, we can only talk about co-occurrence rather than causation. Nevertheless, NDDs are associated with higher rates of incontinence in children and adolescents, including nocturnal enuresis and daytime urinary incontinence [99]. In a small-scale study, urinary incontinence was observed in 85.1% of adults (out of 27) and 90% of children/teens (out of 20), and ASD patients had high prevalence of nocturnal enuresis [100]. Moreover, there is a possible association between kidney diseases and ASD as well [101,102]. Interestingly, in *Caenorhabditis elegans*, the ASD-related NLGN gene was found to also be important in osmoregulation [103] (Table 2). However, in NLGN3 KO mice, the OTergic system was found to be implicated in ASD-like symptoms [104]. In mice, heterozygous TSHZ3 +/− haploinsufficiency resulted in both ASD and renal abnormalities [105] (Table 2). The previously mentioned common genetic variants of the angiotensin receptor 2 may also contribute to urinary problems in ASD with a possible involvement of the VPergic system [15]. 

VP receptors are expressed in the liver as well [106], where the glycogen synthase produces glycogen to store energy [107]. Indeed, the glycogen synthase kinase 3β, one of the modulators, has been implicated in ASD [108], so much so that its inhibitors were even suggested for therapy [109]. VP resistance is observed in poorly controlled non-insulin-dependent diabetes mellitus subjects, which might contribute to their lower plasma volume [110]. Glucose transporter 3 (GLUT3) was coregulated in the neurohypophysis with VP [111] and its deficiency was also implicated in ASD, leading to social and communication problems and stereotyped behavior [112] (Table 2). In line, in V_1a_ receptor KO mice, impaired glucose homeostasis was found [110]. 

VP might influence gut secretion [113]; however, gut microbiome can influence osmoregulation (at least in gerbils [114]). As the microbiome shapes—among others—the social behavior of the animals at several points from olfaction (with VP role [115,116]) to direct effect on social brain and social signaling molecules, e.g., VP-OT [117], we might assume a trilateral cooperation between VP, microbiome and ASD.

The coagulation pathways might also be altered in ASD [118,119]. Interestingly, desmopressin (1-desamino-8-D-arginine vasopressin, DDAVP), a V_2_ agonist, is widely used for promoting coagulation among others in von Willebrand disease (first line treatment) [120] and hemophilia [121]. 

However, all these peripheral VP-ASD connections are rather speculative and need further confirmations. Moreover, in the SHANK3 KO rat model, the plasma VP levels were normal [122]. Therefore, we concentrated on the central nervous system (CNS).

#### 3.2.2. Social Behavior and Vasopressin with Implication in Autism

Impairments in social behavior are one of the main characteristics of ASD (Figure 3). Interestingly, both VP and OT have been connected to it and may regulate social behavior at the genetic, circulatory, neural functioning, and pharmacological levels [123]. Indeed, existing evidence suggests that VP can influence social functions already at the perceptional level and influence how sensory information is interpreted [124]. In this regard, the importance of VP in olfactory function was also confirmed before [115,116]. Moreover, VP was also found in the retina [125], VPergic fibers may project to the suprachiasmatic nucleus (SCN), the known center of circadian regulations (see later) [126,127].

Several animal models of ASD exist, where the contribution of VP was supposed mainly upon their effect on the social domain (Table 2). In the following section, we tried to discuss major findings along different phyla/species. 

**Table 2:** Animal models of autism with possible contribution of vasopressin.

Model			Major Problems	References
Type	Name/Implicated Molecule			
Genetic models	KO	OTR	soc.	[57]
		CNTNAP2	soc., com.	[128]
		MAGEL2	soc.	[129]
		OPRM1	soc.	[130,131]
		Klf7	soc., rep.	[132]
	Fragile X	FMR1	soc., rep., motor problem, mood	[40]
	Rett syndrome	MECP2	soc., com.	[133]
	Tuberous sclerosis	TSC1, TSC2	soc., rep.; cerebellum; V2 antagonist	[134]
	Indirect evidence	NLGN mutations	soc., rest., com.	[103,135]
		TSHZ3 KO	soc., rep., narrowness of the field of interest	[68,105]
		GLUT3 KO	soc., rep., com., memory problems	[111,112]
		parvalbumin KO	soc., rep., com.	[136,137]
		GAP43	soc., resistance to change	[138,139]
		SERT variants	soc., rep.	[140,141,142,143]
Environmental models	Drugs	VPA	soc., rep., com.	[144,145]
	Maternal infection and inflammation	poly I:C	soc., rep.	[146,147]
		LPS	soc.	[148,149]
		MIA	soc.	[147]

Abbreviations: CNTNAP2: Contactin Associated Protein 2; Com: communication problems; Fragile Mental Retardation 1 locus (FMR1); GAP43: synaptic growth-associated protein-43; GLUT3: neuronal glucose transporter isoform 3; klf7: Krüppel-like factor 7; KO: knockout; LPS: lipopolysaccharide; MAGEL2: Melanoma Antigen Gene Family Member L2; MIA: maternal immune activation; methyl-CpG binding protein 2 (MECP2), NLGN: neuroligin; rep: repetitive behavior; poly I:C: polyriboinosinic: polyribocytidylic acid; OPRM1: μ opioid receptor; soc: social problems; TSC: tuberous sclerosis complex; TSHZ3: a zinc-finger transcription factor; VPA: valproate [59].

The VP-OT peptide affects social behavior already in ants [150]. Zebrafish (*Danio rerio*) is another good model for social behavior [66]. In relation to VP, after an aggressive encounter, the VP/vasotocin levels were higher in most brain areas of the winning zebrafish than in the losing ones. Moreover, fishes lacking OTRs showed antisocial-like behavior by the age of 8 weeks post fertilization [151]. A similar role can be identified in birds: pair bonding in zebra finches elevated their immunopositive V_1a_ receptor numbers of selected brain areas [152]. VP/vasotocin was also critical for vocal learning in birds during development, a form of social communication [125]. In hamsters, it is theorized that VP serves as an ancestral molecule in scent marking and consequent territorial behaviors such as pair bonding [153,154,155]. 

Other studies showed decreased aggression after VP treatment in male mice reared in social isolation [156]. Mice are also widely used to study the effects of genetic alterations. In relation to ASD, direct manipulation (overexpression or knocking out (KO)) of the V_1a_ gene may influence social behavioral outcomes [41]. Moreover, VP treatment has been shown to ameliorate social deficit via V_1a_ receptors in OTR-deficient mice [57] (Table 2). CNTNAP2 is a key gene implicated in the manifestation of ASD symptoms, particularly in language disabilities (Table 2). In CNTNAP2-deficient mice, social deficits were improved after VP administration [128]. However, this effect was found to be mediated by OTRs, and not by V_1a_ receptors. On the other hand, a deficiency of MAGEL2, a candidate gene for ASD and Prader Willi Syndrome, resulted in impaired social adaptation and discriminative social exploration caused by diminished VP, but not OT signaling in the lateral septum (LS) [129] (Table 2). μ opioid receptor (OPRM1)—possibly through a connection with the V_1a_ receptors [130]—was also implicated in ASD with social motivation and skill problems in KO mice [131] (Table 2). Parvalbumin KO mice exhibited several ASD-like symptoms including social interactions and communication deficits [136] (Table 2). Linked to VP, developmentally, the V_1a_ receptor modulates the number of parvalbumin positive interneurons in the cortex [137]. Among drug-induced ASD models, maternal viral infection (named maternal immune activation, MIA) can be mimicked via polyriboinosinic–polyribocytidylic acid (poly I:C) administration, which, in mice, induced changes in V_1a_ mRNA expression in the hypothalamus of the offspring [147]. On the other hand, the bacterial infection model lipopolysaccharide (LPS) elevated maternal VP expression [157].

In rats, maternal aggression was also tied to VP, as high-anxiety-related behavior (HAB, based on elevated plus maze behavior) animals have an increased VP mRNA expression in PVN and limbic areas accompanied by increased maternal aggression putatively mediated by V_1a_ receptors [158]. However, V_1b_ receptors in the CA2 hippocampal region were also implicated in aggression [63,64]. One of the most famous rat KO models is the Brattleboro, which has a naturally occurring single-nucleotide deletion in the VP gene (exon 2), resulting in an inactive VP precursor. Thus, they have diabetes insipidus and urinate excessively due to missing the peripheral VP hormone. Moreover, they also exhibit behavioral alterations in social behavior, cognition [159,160] and stress response [159,161,162,163], making them an ideal model for SCZ [164,165,166]. However, during early development, they can serve as an ASD model as well. Indeed, we suggest that their social communication deficit during maternal-separation-induced ultrasound vocalization (MS-USV, Table 1) might provide a good test for new ASD treatment [167]. Based upon a KO mice model [168], as well as antagonist treatment in rat pups [169], V_1b_ receptors might be responsible for this reduced communication. However, the reduced MS-USV was suggested to be a sign of anxiolysis. Nevertheless, maternal neglect of Brattleboro rats [170] might contribute to the disturbed development of the offspring, which might lead to the development of ASD-like symptoms in them [171]. Indeed, V_1a_ receptor was associated with human maternal behavior [172]. In a VPA-induced ASD model, the subcutaneous (s.c.) administration of VP to adolescent rats alleviated social preference deficits and stereotyped behaviors, in parallel with an increase in cerebrospinal fluid VP concentration [173]. Moreover, in a rat MIA model with poly I:C administration, a reduced maternal VP level was found [146].

The role of VP in social behavior was also confirmed in an outdoor, ecologically relevant context, in free-living Richardson’s ground squirrels (*Urocitellus richardsonii*) [174]. In this species, chronic s.c. VP administration via an osmotic minipump increased male social vocalization and decreased their social aggression, thus supporting its pro-social role.

Despite general belief of the V_1a_ receptor’s contribution to social behavior [175], in a study based on more than 3000 h of observation of 201 *Rhesus macaques,* the common genetic variation in the V_1a_ receptor gene was not responsible for their social behavior [176]. Moreover, a recently developed V_1a_ receptor KO hamster strain showed enhanced rather than reduced social communication, although this effect might be confounded by compensation in other systems [177]. Thus, we might assume some role of the V_1b_ receptor as well in, e.g., maternal behavior [178]. Indeed, in humans, the V_1b_ receptor participates in emotional empathy and social behavior [179]. Carriers of the G allele of V_1b_ single-nucleotide polymorphism (SNP) rs28373064 have been reported to be more empathetic and pro-social [145]. Furthermore, the rs35369693 and rs28632197 SNPs of V_1b_ were associated with ASD [180]. However, the variations in V_1a_ receptors have been also significantly linked to ASD in humans [180]. Studies showed that rs11174815, rs7294536, rs3759292, and rs10877969 SNPs of the V_1a_ receptor were correlated with ASD [181,182]. Moreover, an interaction between OPRM1, V_1a_ receptor and social behavior was confirmed in students [130].

All in all, the aforementioned data clearly indicate VPergic contribution to social problems in ASD with V_1a_ receptor implication. However, more human data are needed, and the sex/gender aspect should also be addressed.

#### 3.2.3. Motor Signs: Repetitive Behavior and Convulsions

Beside social communication problems, alterations in motor function (especially stereotyped, repetitive behavior) are further core symptoms of ASD (see earlier), and vasopressin can also play a central role in the appearance of these.

##### Grooming

Already in 1981, the allo-grooming- and scratching-inducing effect of intracerebroventricular (i.c.v.), but not peripheral administration of a posterior pituitary extract was described in mice [183] (Figure 2). This short effect was not due to vasoconstriction [184], but was related to the vasoconstrictor properties of the analogues, suggesting the involvement of V_1a_ receptors [185]. The grooming-inducing effect of low dose (below 100 pg) i.c.v. VP was also confirmed in rats, together with an inhibitory effect on exploration [186]. Later rat studies found even larger doses (30 ng) to be effective [187]. In hamster, both peripheral and central (intra-SCN) injection promoted grooming [188]. In line with this, the male [189], but not female [190], VP-deficient Brattleboro rat strain also showed reduced grooming, and in male squirrel monkeys, i.c.v. VP administration increased both grooming and scent-marking stereotyped behaviors [191]. However, this effect was not specific to VP, as a similar dose of OT could also elicit grooming both in male and female rats [192].

In contrast, in male rats, peripheral s.c. VP administration reduced grooming in open-field conditions 15 min, but not 60 min, after its administration [193]. A subsequent study found arginine VP (major form in humans and rodents) to be ineffective, while lysine VP had a similar reducing effect on grooming [194]. These discrepancies might be easily explained by the different peripheral and central role and receptor repertoire of VP.

##### Marble Burying

Marble burying might also reflect a repetitive behavior (Figure 3), often modeling OCD [195]. Although we could not detect any genotype difference in female VP-deficient Brattleboro rats [190], male Brattleboro and V_1a_ KO mice buried fewer marbles than the controls [196,197], an effect not influenced by manipulation of the peripheral V_2_ receptors [197]. Moreover, during the adolescent period, both male and female Brattleboro rats displayed reduced marble burying [198]. However, these alterations were interpreted as anxiolysis and not reduced repetitive behavior.

##### Epilepsy—A Comorbidity

Nearly one-half of the individuals diagnosed with ASD have also been diagnosed with comorbid epilepsy [199]. Especially important, temporal lobe epilepsy (TLE) is highly prevalent, observable in one-third of ASD patients [200]. This might explain unpredictable emotional outbursts, hypersensitivity and hyperreactivity to trifling noises; thus, not only motor symptoms will occur.

I.c.v. administration of VP into rats induced dose-dependent (1–10 ng/rat) barrel rotations, a violent and apparently uncontrolled motor activity, suggesting a connection between VP and epilepsy [186]. Furthermore, VP administered s.c. (1 and 3 μg/rat) potentiated pilocarpine-induced seizures [201]. Even in febrile seizures (see later via thermoregulation), high VP doses had a pro-convulsant effect [202]. Thus, it was generally considered that VP is a pro-convulsant [175,202]. In accordance, antiepileptic drugs, including VPA, reduced VP-induced barrel rotation [203], without influencing serum VP levels [204]. Interestingly, the peripheral (intraperitoneal, i.p.) administration of low dose VP (0.1 μg/kg) lowered the pentylenetetrazol-induced seizures threshold, thus being pro-convulsant, while higher doses (10 and 20 μg/kg) increased it, thus being anti-convulsant [205]. The pro-convulsant effect was antagonized by both the V_1a_ and the V_1b_ antagonists, as well as the V_2_ antagonist, while only the V_1b_ (and OTR) antagonist prevented the anti-convulsant action [175,201,205]. This suggests an atypical receptor activation of the higher doses, and a putative endogenous pro-convulsant effect of VP.

##### Stress in Autism—The Third “Hit”

Despite atypical ANS and HPA functioning of ASD patients both at resting state and during the presence of social and/or non-social stressors being generally accepted [30], there are still some controversies.

- The Autonomic Nervous System

For the correct interpretation of emotions, feedback from the ANS is essential as formulated by the often debated James–Lange theory [206]. Indeed, recent studies examining the heart rate variability confirmed dysregulation of the ANS in adult [207] and adolescent [208] ASD patients with reduced parasympathetic and increased sympathetic activity. The dysregulation of the ANS might lead to both hypo- and hyperarousal [209]. As social abilities are optimal when arousal is normal, when arousal increases due to misinterpretation of danger signals from the environment, social behaviors are compromised. Thus, ANS problems may contribute to socio-communication deficits in ASD.

Although VP and ANS were mostly examined separately in ASD, based on the cardiovascular peptide nature of VP and its strong interaction with ANS [210], we might assume a trilateral collaboration between VP, ANS and ASD. Indeed, VP is considered to be part of the “extended” ANS [211].

However, some authors argue that the reported ANS dysfunction in ASD patients is confounded by high anxiety related to the study situation or might be due to other comorbidities [212]. Thus, the question remains open.

- Vasopressin in Thermoregulation with Implication in Autism

Thermoregulation is accomplished via autonomic and behavioral responses controlled by the ANS [213].

There is no evidence to suggest that individuals with ASD are more prone to fevers than others. However, fever management may be more challenging in them due to their sensory hyper-sensitivity [214].

According to some observations, children with ASD show improved communication and social behavior during their febrile episodes [215,216]. The mechanism behind this has not yet been fully elucidated. Nevertheless, VP is involved in thermoregulation during fever: an early study found that central VP release increases during fever in sheep [217]. This phenomenon was later supported by a human study: plasma and cerebrospinal fluid VP concentrations were found to be elevated in febrile individuals compared to those in controls [218]. In the literature, VP is also referred to as an endogenous antipyretic: it influences thermoregulatory neurons in the anterior hypothalamus, preoptic, and septal areas [219,220] and can participate in tolerance to pyrogens in these areas [221]. However, in rabbits, the contribution of the peripheral VP effect through the V_1_ receptors was also suggested [222]. Thus, VP signaling might contribute to the transient beneficial effect of febrile episodes in ASD.

However, despite a beneficial antipyretic effect, high levels of VP might even induce febrile convulsions [202]. Yet, the receptor specificity is still questionable.

- The Hypothalamic–Pituitary–Adrenocortical Axis

It is textbook knowledge, that—together with corticotropin-releasing hormone (CRH)—VP from the PVN is a major regulator of the HPA axis, stimulating the ACTH secretion in the anterior lobe of the pituitary (AL) through V_1b_ receptors [163,220]. There are reports on blunted [223] or exaggerated [224,225] cortisol (the end-hormone of the HPA axis) release in ASD patients in response to ACTH or perceived stressors with high individual variability [226]. Besides methodological differences (note that the measured stress-hormone levels are very sensitive to the sampling methods), the selective contribution of central–not only AL-V_1b_–receptors might shade the picture resulting in stressor-specific effects.

The synaptic growth-associated protein-43 (GAP43), an ASD candidate gene of interest, is deeply involved in the regeneration of VP production after injury [138], and, at the same time, its deficiency in GAP43 KO mice leads to resistance to changes as well as to stress vulnerability [139], further supporting VPerg’s contribution to an atypical ASD stress response (Table 2). We might even assume that altered stress reactivity designates a subpopulation of ASD patients as sensitive to VPergic manipulations.

- Mood Disorders and Autism—A Comorbidity

A meta-analysis conducted in 2019 showed that in adult ASD patients, the current prevalence of anxiety disorders was 27% (in comparison to 19.1% in a normal population), while that of depressive disorders was 23% (compared to 5% among neurotypical adults) [33,34]. VP was considered an ‘endogenous anxiogenic/depressogenic substance’ [220] based upon its prevalent role in HPA regulation and the stress-related nature of anxiety and depression [161,227].

- Anxiety

In a rodent model of anxiety (HAB and low anxiety behavior (LAB) mice and rats, see earlier), the VP gene was found to be the candidate gene for inborn anxiety [228]. In line with this, the VP-deficient male Brattleboro rats showed reduced anxiety- and depression-like symptoms [189]. Furthermore, the V_1a_ receptor KO mice exhibited reduced anxiety [196,229] and the V_1a_ receptor antagonist was found to be anxiolytic [230]. These results support the theory that the V_1a_ receptor subtype plays an important role in the regulation of anxiety-like behavior. In support, a newly developed V_1a_ receptor antagonist reduced anxiety-potentiated startle independently of fear-potentiated startle in healthy volunteers [231].

At present, we might only assume that VP also contributes to anxiety in ASD, which raises the possibility of a V_1a_ antagonist treatment. However, its systemic administration might be questionable as V_1a_ receptors are present in blood vessels; thus, these antagonists might lead to vasodilatation and hypotension.

- Depression and Serotonin

In relation to depression, many preclinical studies supported the involvement of the V_1b_ receptor, the major regulator of the HPA axis in the development of the symptoms [220,232]. However, clinical studies did not support this notion [233]; therefore, most investigations following this direction were suspended [234]. For now, we can assume that V_1b_ antagonists might be effective only in a selected subpopulation or gender.

On the other hand, serotonin is highly implicated in depression, as presently prescribed drugs are mainly selective serotonin reuptake inhibitors (SSRI). On the other hand, an average of a 50% increase was found in plasma-based serotonin levels in one-third of ASD individuals, supporting the connection between serotonin and ASD [235]. This increased peripheral level is thought to reduce central serotonin function due to negative feedback [200]. Moreover, genetic variation in the serotonin transporter (SERT) in mice led to—among others—ASD-like behavioral changes [140,141] (Table 2). However, this is a rather complex topic and different players of the serotoninergic systems might be differentially implicated [236]. In terms of the role of VP, abnormal HPA axis function might lead to mood disorders or even to suicidal behavior via the dysregulation of the serotonergic system [142]. There is a strong interaction between VP and serotonin in social contexts, modulating the appearance of aggressive behavior [143]. Indeed, serotonin might antagonize VP activity in the CNS [237,238]. In hamsters, VP induced repetitive aggressive behaviors, which were inhibited by the simultaneous use of a serotonin (5HT_1a_) agonist [239]. Thus, VP might contribute to the effectiveness of SSRIs in ASD (see later).

##### Sleep Disturbances in Autism—Another Third “Hit”?

One of the most frequent comorbidities in patients with ASD are sleep disorders (prevalence between 50% and 83%) [240,241], which contribute to a decreased quality of life [242]. Indeed, problems in the development of the sleep–wake rhythm might deeply contribute to the appearance of the NDDs [243].

In the literature, VP has been associated with sleep mostly in relation to mood disorders [244], but not to ASD. Nevertheless, VP is deeply implicated in circadian regulation (see Section 3.3.3), which might also be disturbed in ASD [245]. Even a causal gene for ASD, the Krüppel-like factor 7 (klf7), a transcription factor in the CNS (Table 2), might induce ASD-like behavior by regulating the circadian rhythm [132], indicating that sleep disturbances in ASD are causes more than they are consequences. Interestingly, besides direct regulatory role in the SCN, the potentiating effect of VP on melatonin secretion in pineal gland was also described in rats [246]. However, it has been disproved in a human study [247].

##### Vasopressinergic Pain Regulation with Implication in Autism

In patients with ASD, perceptional disturbances are common and hypersensitivity to pain might also occur [248]. Interestingly, more recent studies suggest that peripheral VP plays a direct role in the regulation of pain [249] and might be used even for postoperative analgesia in humans [250]. Furthermore, the V_1a_ receptor subtype has been described in mice on the dorsal root ganglia, the major sensory ganglion, which might contribute to the analgesic effect of VP (as well as OT) [251].

However, another study suggested that VP modulates pain perception through brain areas outside the pain matrix [252], at least in rats [253]. In support, pain might increase the expression of VP in the rat PVN, suggesting an increased stress state [254]. We might assume that alterations in the cerebrospinal fluid VP content described in ASD patients might also contribute to their pain hypersensitivity, possibly through the PVN [255].

Although there are mainly rodent studies in connection with pain, we can hypothesize that evolutionarily well-conserved signaling pathways also regulate pain sensitivity in humans with VP contribution.

### 3.3. Brain Areas as Possible Links between Vasopressin and Autism

Considering the aforementioned wide range of processes, VP might influence the development of ASD (or ASD-like) symptoms at several points. We tried to summarize the available literature on brain areas with VPergic contribution along the two major domains (social skills and repetitive behavior) and added stress and circadian regulation as possible third “hits”.

#### 3.3.1. Social Behavioral Network

**Figure 5:**
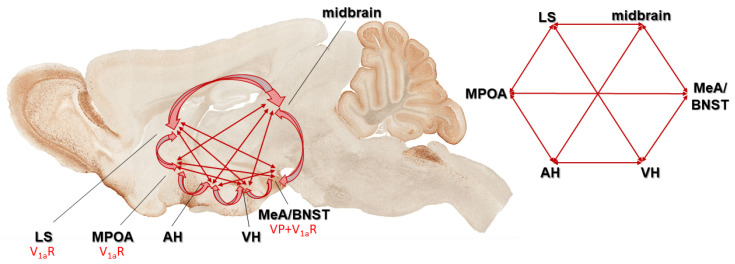
According to the hypothesis of Newman, these brain areas form an integrated social behavior circuit, a subcortical limbic network (social behavior neural network (SBNN)) that subserves the entire spectrum of social behaviors. All these areas have been identified as an important place of activation or regulation of more than one social behavior and each is reciprocally interconnected with all others. Vasopressin (VP) and its V_1a_ receptor can be found on many parts of this network. Abbreviations: AH: anterior hypothalamus; BNST: bed nucleus of stria terminalis; LS: lateral septum; MeA: medial part of the amygdala; MPOA: medial preoptic area; VH: ventromedial hypothalamus.

The signaling mechanisms behind the social role of VP have been studied well. Indeed, VP receptors have been found in brain areas of the social behavior neural network (SBNN) [124,256] as well as in the mesocorticolimbic dopamine system [257]. These networks control social behavior in mammals according to the hypothesis of Newman [256] (Figure 5).

The medial preoptic area (MPOA)—part of the SBNN—might have a role in regulating social communication and social touch. In this context, MPOA-V_1a_ receptors were found to be important in the olfactory communication in hamsters, called flank marking [153,258]. Recently, a thalamo-preoptic pathway was found to regulate social touch in female rats, with an existing human analogue [259]. As VP is released from MPOA [260] and regulates maternal care, we might assume its involvement in this process as well.

Another SBNN area, the lateral septum (LS), seems to have the most dominant role in regulating social recognition and social memory through VP [261,262,263]. Re-expressing V_1a_ in the LS of KO mice normalized, while the overexpression in WT increased social memory [261]. Furthermore, in prairie vole (*Microtus ochrogaster*), the most studied social animal model, the septal VP fiber density showed alterations according to the paternal behavior, and V_1a_ antagonist reduced the appearance of paternal responsiveness [155]. As a part of the paternal repertoire, the grooming of offspring was also elicited by LS injection of VP [155]. Septal VP may underlie the species differences and different life strategies of monogamous prairie vole and the polygamous montane vole (*Microtus montanus*) as well [264]. In a monogamous mice species (*Peromyscus californicus*), more VP receptors were found in the LS compared to a polygamous one (*Peromyscus maniculatus*) [265]. A recent study in an animal model of ASD has described a direct relationship between LS, VP, and social behavior [266]. A similar suggestion can be made based on a placebo-controlled study in healthy adult volunteers, as intranasal (i.n.) VP administration increased LS activity while viewing facial photographs [267].

In mice, the medial part of the amygdala (MeA) receives VP innervation [268], but it contains VP producing neurons as well and is implicated in the regulation of social behavior [269]. Moreover, VP was able to alter VPA-induced genetic alterations in the amygdala [144]. A human functional magnetic resonance imaging (fMRI) study suggested that VP modulates prefrontal cortex (PFC)-amygdala circuitry during emotion processing using facial emotion recognition [270]. Furthermore, in 3–5-year-old ASD children, negative functional connectivity of left amygdala and left supramarginal gyrus was accompanied by lower plasma VP levels; however, this was only detectable in boys [271]. In addition, an increased volume of the left amygdala has been found in children with ASD, and this was positively correlated with plasma VP levels. As a possible background, the amygdala of ASD patients showed an initial increase in the number of mature neurons followed by a decline into adulthood compared to healthy controls [272].

The extended amygdala region, the bed nucleus of stria terminalis (BNST) [273], also contains VP-producing cells [274]. Among others, BNST is associated with anxiety, addiction [275], and an innate fear response [276]. Moreover, a 2019 human study described it as one of the central brain areas of social anxiety [277]. Investigations on juvenile rats showed that VP is important in social play, and the PVN and BNST VP systems, in particular, regulate this behavior [274]. In the VPA model of ASD, VPergic BNST projection was significantly altered [278]. Moreover, maternal LPS injection-induced reduction in juvenile play was accompanied by reduced VP mRNA content here as well as in the MeA [149].

The PFC plays a pivotal role in social interactions, serving as a hub for motivation, affiliation, empathy, and social hierarchy [279]. In maternal VPA-treated monogamous prairie voles, PFC expressed less V_1a_ receptor mRNA together with reduced sociability [280]. Additionally, VP altered VPA-induced genetic alterations in the PFC [281].

An often-neglected brain area is the olfactory bulb, containing VP as well [115,116]. Indeed, olfactory bulb dysgenesis was described in ASD and—according to the theory of Brang and Ramachandran [200]—might even be causally involved in mirror neuron system malfunction.

There are interactions between the above-mentioned areas, as LS receives VPergic innervations from BNST and MeA, forming a regulatory network for shaping social behavior [282,283,284]. The role of VP in aggression, a specific social behavior often observable in ASD patients, is brain-area-specific as its release in the LS facilitates, while BNST decreases, intermale aggression in rats [285]. Moreover, a fine balance exists between different areas, as SON replacement of VP in Brattleboro rats led to an increase in friendly interactions, which were originally normal in KO animals [159]. The VP immunoreactivity in both the LS and BNST is sexually dimorphic, and, in males, it is dependent on neonatal testosterone levels, later shaping aggressive behavior in mice [286]. In hamsters, VP injections to the ventrolateral hypothalamus facilitated aggression, an effect which was antagonized by i.p. SSRI administration [237].

Maternal behavior is another interesting social behavioral type, strongly influencing infant development, where VP is also deeply implicated [172]. Interestingly, MPOA administration of V_1b_ antagonist increased, while its BNST injection decreased offspring care [178]. However, it is mostly the LS V_1a_ receptors that are assumed to be involved in this process [287].

In summary, the dysfunction of the social behavioral network occurring during ASD might be related to VP at many points, among which the role of LS is best clarified.

#### 3.3.2. Motor Behavior and Vasopressin

The previously mentioned flank marking in hamsters is, in fact, a stereotyped behavior, which was elicited by VP injection into the MPOA [288] and was antagonized by the V_1_ antagonist in the LS and BNST [154]. Furthermore, periaqueductal grey (PAG) injections of VP were also able to elicit this behavior both in male and female hamsters [289]. Moreover, SCN injection of VP in hamsters reduced spontaneous nocturnal running [188].

Interestingly, a recent study using optogenetic technique in mice elicited immediate grooming via the stimulation of the PVN VPergic cells [290]. On the other hand, grooming was elicited via amygdalar VP injection in male rats as well [291,292]. However, a repeated injection induced not only grooming, but also barrel rotations and myoclonic/myotonic-like convulsive behavior [293].

Barrel rotation was also induced by nodular cerebellum VP injections [294]. Indeed, cerebellum has attracted renewed interest as a brain area at the crossroads of cognitive and motor symptoms characteristic of ASD [295]. The cerebellum is not only critical for the coordination and adjustment of movement but is also involved in higher functions such as cognition, speech and emotion, all of which are altered in ASD [296]. Of interest is the fact that perinatal cerebellar injury is the highest risk factor for ASD, apart from an affected monozygotic twin [22,297]. In the tuberous sclerosis model of ASD, Purkinje cell damage was associated with ASD-like symptoms [298]. VP administration increased cerebellar activation to infant cry, supporting its contribution to emotional regulation [299].

The hippocampus is a prominent source of epileptic seizures. It contains both V_1a_ and V_1b_ (as well as OTR) receptors and both were shown to contribute to hippocampal excitability [300]. Moreover, the magnocellular PVN and SON, as well as parvocellular BNST and amygdala, project to the hippocampus.

#### 3.3.3. Vasopressinergic Link to Stress, and Related Disorder

The anxiolytic effect of VP is presumably connected to V_1a_ receptors localized in BNST [301]. Moreover, its effectiveness, similar to that of SSRIs, may be due to a BNST-derived VP-ergic innervation into the dorsal raphe nucleus (one of the main serotoninergic nuclei), but this pathway has only been described in mice to date.

On the other hand, the depressive-like behavior of HAB rats was accompanied by the overexpression of the VP gene in the PVN [302]. Differences in PVN have also been reported in human depressed patients compared to healthy controls, where both the VP genes and the V_1a_ receptor were overexpressed [303,304].

PVN is also a major regulator of the ANS, being a concertmaster and providing a place for VP-ANS-ASD interaction [210,305].

#### 3.3.4. Circadian Rhythm and Vasopressin

Electroencephalography (EEG) studies have suggested that ASD patients show alterations in rapid eye movement (REM) sleep [306], possibly due to a disturbance of the SCN, the center of endogenous clock. Indeed, in the VPA-induced animal model of autism, circadian dysregulation was found due to alterations within the core clockwork of SCN [307].

One of the first discovered neurotransmitters of the SCN was VP, confirmed later in many species, including humans [308]. The neuronal activity of VPergic cells of the SCN shows daily variation and may control neuroendocrine (stress and gonadal axis) and other (e.g., sleep–wake) rhythmic changes [309]. Research on hamsters showed that SCN VP cells might possibly respond to melatonin signals [310]. Furthermore, in rats, VP administration into the PVN elevated the plasma melatonin levels [311]. This might strengthen the hypothesis that VP can mediate circadian responses to melatonin in the SCN. Investigations into melatoninergic VP regulation in the human SCN may pave the way for new perspectives to understand the mechanism of sleep disturbances in ASD [312].

**Figure 6:**
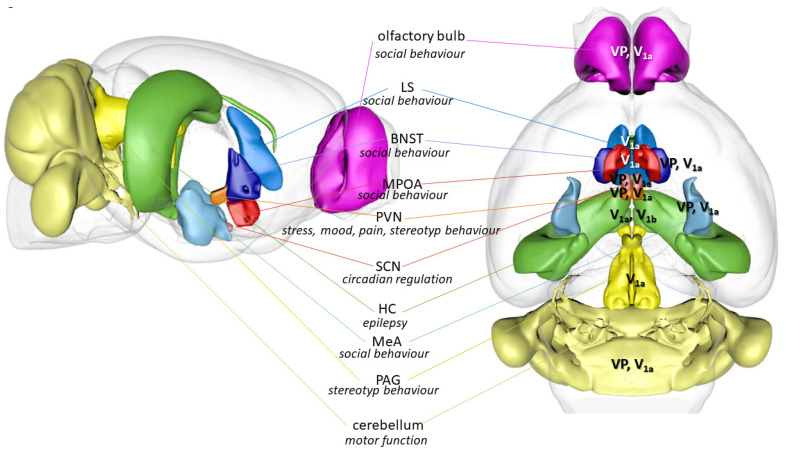
Brain areas containing vasopressin producing neurons or vasopressin receptors with possible involvement in autistic behavior. Major role of the nucleus is given. Abbreviations: BNST: bed nucleus of stria terminalis; HC: hippocampus; LS: lateral septum; MeA: medial amygdala; MPOA: medial preoptic area; PAG: periaqueductal grey; PVN: paraventricular nucleus of the hypothalamus; SCN: suprachiasmatic nucleus; V_1a_: vasopressin receptor; V_1b_: vasopressin receptor; VP: vasopressin producing cells (based upon a mouse brain in https://scalablebrainatlas.incf.org/composer/index.php, accessed on 1 August 2023).

All in all, VP may play a key role in the onset of ASD symptoms and comorbidities in several brain areas, which can be different depending on the symptoms. In fact, ASD is a network disorder; thus, highlighting an area in particular would be difficult. For a summary of the possible brain areas with VPergic contributions to ASD symptoms, see Figure 6.

## 4. Sex Differences in Autism with Focus on Vasopressin

The higher prevalence of ASD in males [313] suggests sex-dependent regulatory processes. However, ASD can be under-diagnosed in females since they can better compensate for the lacking social skills or might show atypical signs [314,315]. Nevertheless, prominent theories have been proposed to explain sex biases, like genetic factors, sex hormones, sociological factors, cognitive differences between the sexes, and environmental insult [316].

In relation to our present topic, differences in the VP and OT system could be one of the underlying causes of the male bias. Since ASD is known as a neurodevelopmental disorder, the disruption of the VP system during early development may play an important role in the pathomechanism disrupting sex-specific neural circuits that are responsible for the sexually dimorphic nature of the social behavior [317]. Excess VP or dysregulation in the VP system could contribute to male vulnerability, while processes mediated via the OT system may explain the resistance in females [318].

In adult rodents, there are gender differences in the amount of VP produced and the density of V_1a_ receptors in specific brain regions [317]. In these regions, the level of VP tends to be higher in male rats and mice than in females [268,283,319]. Of note, the LS of adult male rats have denser VP fibers but fewer V_1a_ bindings than females [320,321]. This is in good agreement with the idea of brain-based sex differences in ASD; however, the authors suggested different neurodevelopmental trajectories leading to the masculinization or feminization of the brain [322]. Indeed, cortico-cerebellar hyperconnectivity was found in ASD females, while hypoconnectivity was found in males [323]. Nevertheless, the effect of VP treatment can also be sex-dependent, as, in free-living, female Richardson’s ground squirrels, it resulted in increased “anxiety-like” behaviors during social challenge, while, in males, it increased social communication and reduced aggression [174].

Alterations in sex hormones may also contribute to sex differences in ASD, as androgen receptors can be found in ASD-related brain regions [316]. On the other hand, sex hormones can affect the levels of VP, and could thus further contribute to male vulnerability in ASD. In the BNST and MeA, more than 90% of the VPergic neurons contain androgen receptors in rats, and VP synthesis was also androgen-dependent in those areas [324]. PVN also has a higher number of androgen receptors in human males compared to females [325]. Testosterone injected in female or neonatally castrated male rats on the first, second or third week of life resulted in an increase in VP fiber density [326]. In contrast, progesterone could inhibit the synthesis of VP in the BNST, the central nucleus of the amygdala and the LS in male rats [327].

In line with this predominant role of VP in males, the VP-deficient male, but not female Brattleboro rats, showed reduced social abilities [166,190]. To date, social deficit has been described in male V_1a_ KO mice only [196,229,261]. In adolescent autistic patients, plasma VP showed negative correlation with repetitive behaviors in boys, while, in girls, the association was positive [328]. In relation to VP-serotonin interaction, gender differences in the response to SSRIs have been also reported [329].

The effects of exogenous VP treatment are also sexually dimorphic and dose-dependent. In prairie voles, i.c.v. low dose VP (0.5 ng) heightened the partner preference in males [330], but had no effect in females [331], further supporting the dominant role of VP in males. However, when 100 ng VP was administered, partner preference was enhanced in both genders [332].

## 5. Vasopressin-Related Possible Therapies in Autism

There is no cure for ASD, and there is currently no medication to treat it. The medications are prescribed mainly to treat self-injury, inability to focus, anxiety and depression (SSRIs), aggression (alpha-2 adrenergic agonist, Clonidine) and hyperactivity (dopamine and noradrenaline stimulant methylphenidate, Ritalin) [333]. Currently, strategies to treat the core symptoms of ASD are directed to correct synaptic dysfunctions, abnormalities in central VP, OT and serotonin neurotransmission, and neuroinflammation [42]. Although, for now, only two antipsychotics (risperidone and aripriprazole) are approved by the American Food and Drug Administration (FDA) for the treatment of some ASD symptoms, there are multiple drugs undergoing active investigation and trials to assess their safety and efficacy, including both VP agonists and—surprisingly—antagonists. However, OT is more intensively studied, even called a pro-social pill [334].

### 5.1. Available Therapies with Possible Vasopressinergic Contribution

Among the most prescribed medications for autism [333], the following VP interactions can be supposed:

From the second-generation antipsychotics used for the treatment of irritability, cariprazine is promising and their serotoninergic effect suggest a possible VPergic contribution [335].

For the improvement of mood, as well as to reduce the frequency and intensity of repetitive behaviors and improve eye contact, SSRIs are often used. In this regard, VP–serotonin interaction might contribute to the possible effectiveness of aggression treatment using SSRIs [238].

As regards methylphenidate (Ritalin), a dopamine (DA) reuptake inhibitor, it is used as a stimulant for the treatment of hyperactivity (paradoxically) and lack of attention in ASD. It was shown that it may influence the VP system [336] and it acts—at least partly—via the V_1a_ receptor [337].

Alpha2-agonist (e.g., Clonidine) may be used for ASD-related hyperactivity, attention deficit, and aggression, and may interact with VP on the cardiovascular function. Indeed, i.c.v. Clonidine administration-induced pressor response was prevented by i.c.v. V_1_ antagonist administration in rats [338]. Interestingly, in humans, Clonidine administration decreased plasma VP levels [339]. In horses, no interaction was found between Clonidine and VP on HPA axis [340]; however, in rats, Clonidine reduced the firing of SON VPergic cells, further supporting an interaction at the level of water balance [341].

As for applied behavior analysis (ABA), in a backtranslation study using ASD model mice, this intervention normalized VP and V_1a_ expression in several brain areas, including MeA [342].

Even transcutaneous electrical acupoint stimulation elevated VP levels in connection with an improvement of ASD symptoms [343].

Although experts do not recommend any specific diets for children (not even gluten- or casein-free), some probiotics might improve gastrointestinal symptoms [344]. As a possible link to VP, in prairie voles, *Limosilactobacillus reuteri* administration resulted in lower anxiety, but also lower social affiliation in female but not male individuals, with a decrease in PVN V_1a_ expression [345].

**Figure 7:**
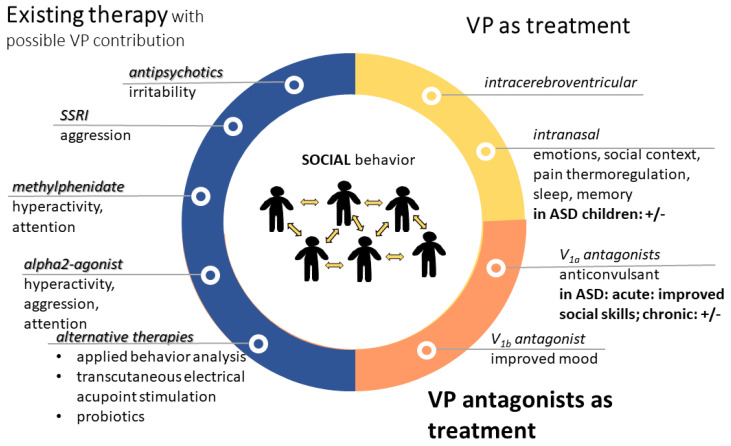
Treatment options in autism with contribution of vasopressin. VP might contribute to the effectiveness of presently available therapies (blue). However, VP alone (yellow) or its antagonists (orange) can be used for therapy. Most of the treatments aim to improve social skills; however, sometimes the results are questionable (+/−). Abbreviations: ASD: autism spectrum disorder; SSRI: selective serotonin reuptake inhibitor; V_1a_: vasopressin 1a receptor; VP: vasopressin.

For a summary, see Figure 7.

### 5.2. Influencing the Vasopressinergic System in Autism-Related Problems

Besides the aforementioned indirect effects, the direct influence on the VP pathway might have therapeutic potential on its own.

As VP does not cross the blood–brain barrier [175], for influencing the central VPergic system, i.c.v. or i.n. application is preferable.

In a rat VPA model, acute i.c.v. VP administration prevented social-interaction-induced brain activation based on blood oxygenation level (BOLD) signal in fMRI [346].

#### 5.2.1. Intranasal Vasopressin Application

In rats, i.n. VP treatment (from PND 21 for 3 weeks) improved maternal VPA injection-induced (E12.5) social deficit, elevated the serum VP level and corrected expression changes related to synaptic and axon dysplasia and oligodendrocyte development in the PFC [281] and amygdala [144].

In male, but not female, marmosets, i.n. VP administration reduced food sharing with increased aggressive vocalization [347]. Accordingly, in monogamous male prairie voles [348], as well as in the coppery titi monkey (*Callicebus cupreus*) [349], a similar treatment reduced partner preference. These preclinical results did not suggest a possible positive effect on ASD symptoms.

However, when VP was administered i.n. for 4 weeks in ASD children aged 6–13 years in a phase 2 randomized clinical trial, improved social responsiveness and social abilities with decreased anxiety and limited repetitive behavior were reported [350]. The response was the strongest in high-plasma VP patients, and depended on the expression pattern of the V_1a_ and OTR receptors. The latter might explain the controversially decreased anxiety, as V_1b_ receptors were more involved in this stress-related disorder. In contrast, a randomized, double-blind, placebo controlled, between-subjects design on 125 undergraduate students (with 41 placebo, 30 females in each), using i.n. VP administration, did not find any effect on social outcomes [351]. In support, i.n. VP administration in rats failed to influence social recognition [352], despite previous effectiveness of the direct olfactory bulb manipulation [116]. Moreover, in healthy male volunteers, i.n. VP administration decreased goal-directed top-down attention control to social salient stimuli with an increase in bottom-up social attentional processing [353]. This effect was similar to OT administration and accompanied by an anxiolytic effect as well. In another study on face processing, a single low-dose i.n. VP (20 IU) administration to men decreased social assessments with a most pronounced effect in V_1a_ risk allele carrier subjects [354]. This suggest that via i.n. application, significant amounts of VP might not reach behaviorally relevant areas in the brain described previously as targets for the central administration of the peptide [352].

For other ASD-related alterations, where we suggested possible VP contribution (Figure 4), the following treatment effects were found:

The activity of brain regions implicated in emotion processing was altered by i.n. VP treatment [41]. In this regard, in humans, i.n. VP regulated the processing of infant cry sounds with emotional contextual information in fathers [299]. In male volunteers, i.n. VP administration increased approaching ratings to some faces, together with increased processing suggested by higher N1 amplitude on the electroencephalograph; however, this effect was highly context-dependent [355]. Another study using fMRI in healthy male subjects reported reduced amygdalar activation to emotional faces after i.n. VP administration [356]. In contrast, another study reported enhanced neural pattern in the right amygdala to social–emotional stimuli observed via MRI [357].

As mentioned before, i.n. VP administration was also able to reduce pain in relation to postoperative orthopedic surgery [250].

Regarding its thermoregulatory role, i.n. VP (more specifically desmopressin, a V_2_ receptor-selective agonist) reduced persisting coldness after brain injury in six patients [358].

In contrast, i.n. VP administration exacerbated physiological ANS parameters in combat veterans [359].

In healthy, elderly subjects, i.n. VP promoted sleep time and improved sleep architecture [360], reinforcing the potential beneficial effect of VP in ASD treatment. However, it was ineffective as regards verbal memory function [361].

#### 5.2.2. Vasopressin Antagonist Treatment

In recent years, vasopressin receptor antagonists have been in the spotlight of drug discovery, especially V_1a_ selective molecules [362]. Publishing Balovaptan as a possible treatment for ASD greatly increased the interest in CNS-acting vasopressin antagonists. Although clinical trials were unsuccessful in many cases, there is still potential in the VP antagonists as shown by several currently ongoing clinical studies.

The main focus is on V_1a_ receptor antagonists. In this context, SRX246, a V_1a_ receptor antagonist, blocked the effect of i.n. VP administration-induced reduced amygdalar activation to angry faces [356]. Moreover, in 2017, a multicenter double-blinded crossover study found that single-dose intravenous (i.v.) infusion of RG7713, a highly selective V_1a_ antagonist in adult males with high-functioning ASD, resulted in a subtle but statistically significant improvement in social communications and social sensitivity [363]. As a follow up, the VP Antagonist to Improve Social Communication in Autism (VANILLA), a double-blinded placebo controlled clinical trial, examined 223 adult men with high-functioning ASD using another selective V_1a_ receptor antagonist, RG7714 (commercially known as Balovaptan) for 3 months [364]. The treatment was well tolerated and resulted in improvement in communication and socialization scores, though not in all aspects of the ASD spectrum (e.g., social responsiveness was not improved). Despite effectiveness during the phase 2 trial [365], in subsequent phase 3 trials in high-functioning children (5–17-year) [366] and adults (above 18-year) [367], the 6-month Balovaptan treatment was ineffective as regards social communication.

Other selective V_1a_ receptor antagonists (like the orally active Relcovaptan) might be effective as regards comorbid epilepsy [175]. On the other hand, for many years, V_1b_ receptor antagonists were developed to treat mood disorders. Despite previous ineffectiveness in major depression [233], V_1b_ receptor antagonists might be effective in subpopulations [368,369] and are therefore still under development (e.g., THY1773 [370], TS-121 [371], ABT-436 [372]). We cannot ignore V_2_ receptors either, as Tolvaptan, a V_2_ antagonist was implicated in the treatment of tuberous sclerosis, a genetic ASD, in a case report [134] (Table 2).

#### 5.2.3. Oxytocin Treatment

As VP might bind to OTRs (see earlier), it is important to note that several animal trials of OT treatment suggested beneficial effects. In children, even a single intranasal OT administration increased the nonverbal information-based judgments [373]. Despite mixed results, a recent meta-analysis found moderate evidence that a 6-week OT treatment might improve the reduced interest and repetitive behavior of ASD children and the effect lasted for at least 6 months [374].

#### 5.2.4. Contradiction

There is an apparent contradiction between the effectiveness of VP as well as its antagonist. A possible explanation can be the age of the participants as well as the method used for drug administration (i.n. for children, other peripheral routes for adults), thereby targeting central or peripheral receptors. Moreover, although VP may stimulate all receptors including OTRs, its effectiveness can be different on them, while antagonists are highly selective, which might shift balance between the VP receptor actions.

## 6. Conclusions

VP as a social hormone with a ubiquitous role might influence the development of ASD symptoms at several points (Figure 4). Although we might even suppose a causative role of VP, at present, only a symptomatic treatment can be assumed. The available treatment options might also influence the VPergic system; however, VP or antagonist administration can also be considered. Controversially, both VP itself and V_1a_ antagonist have already been proven to ameliorate several symptoms (Figure 7). This discrepancy drew our attention to the possibility of subpopulation (i.e., stress-sensitive, male individuals) and/or brain-area-specific manipulation, which was not emphasized before. One may suppose that ASD patients with low blood VP level might benefit more from VP therapy [350,375], and the doses should thus be adjusted accordingly. Although, in humans, systematic treatments are preferable, viral vectors are widely used in therapy nowadays to provide a possible life-long treatment with a single injection [376]. However, it would not be easy to dissect a certain brain area, as the role of VP in socioemotional functioning recruits multiple brain networks distributed across the whole brain [377] (Figure 5 and Figure 6). As the aforementioned clinical studies have limitations (e.g., low sample size, male-biased sample, short treatment duration, not medication-free patients [350]), further preclinical and clinical trials are needed.

## Data Availability

Not applicable.

## Supplementary Materials

The following supporting information can be downloaded at: https://www.mdpi.com/article/10.3390/biomedicines11102603/s1, Figure S1: Statistical data about the importance of autism spectrum disorder (ASD) [1,2,3,378,379,380].
