# Supplementary material for: Vasopressin as Possible Treatment Option in Autism Spectrum Disorder
Source: Biomedicines. 2023 Sep 22;11(10):2603. doi: 10.3390/biomedicines11102603 (PMC10603886; doi:10.3390/biomedicines11102603)
Supplement: Supplementary file 1 [file biomedicines-11-02603-s001.zip › biomedicines-2579594-supplementary.pdf]

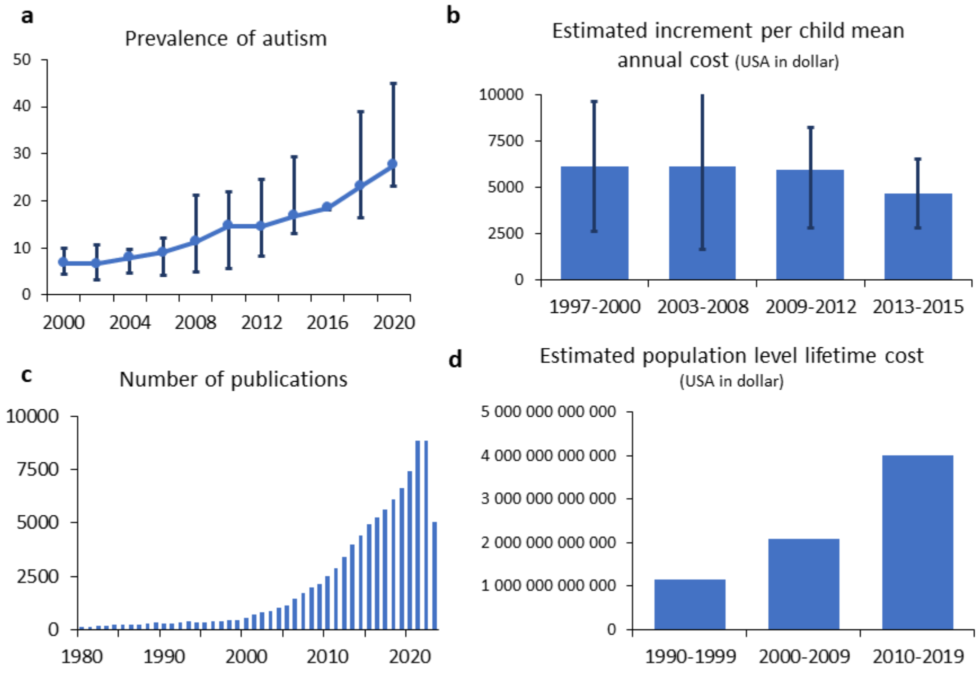

Supplementary Figure S1. Statistical data about the importance of autism spectrum disorder (ASD). (**a**) The prevalence (per 1,000; in 8-year-old children) based upon the data of Center for Disease Control and Prevention, USA [1-3]. It increased from 4.5 per 10.000 children in 1966 with a 2.6:1 boy to girl ratio [4] to 18.5 per 1,000 (one in 54) children aged 8 years in 2016 with 4.3:1 boy to girl ratio [2], showing an annual growth rate of 0.42% [1]. (**b**) The incremental annual per-child cost (USD) of ASD [5, 6]. (**c**) Increasing appearance of ASD in PubMed search with keywords „autism" or „ASD" and „date of publication". (**d**) Estimated lifetime cost of ASD in USA [7]. Whiskers represent interval.

References

1. Feng, X. W.; Hadizadeh, M.; Cheong, J. P. G., Global Trends in Physical-Activity Research of Autism: Bibliometric Analysis Based on the Web of Science Database (1980-2021). *Int J Environ Res Public Health* **2022,** 19, (12).
2. Maenner, M. J.; Shaw, K. A.; Baio, J.; EdS; Washington, A.; Patrick, M.; DiRienzo, M.; Christensen, D. L.; Wiggins, L. D.; Pettygrove, S.; Andrews, J. G.; Lopez, M.; Hudson, A.; Baroud, T.; Schwenk, Y.; White, T.; Rosenberg, C. R.; Lee, L. C.; Harrington, R. A.; Huston, M.; Hewitt, A.; PhD; Esler, A.; Hall-Lande, J.; Poynter, J. N.; Hallas-Muchow, L.; Constantino, J. N.; Fitzgerald, R. T.; Zahorodny, W.; Shenouda, J.; Daniels, J. L.; Warren, Z.; Vehorn, A.; Salinas, A.; Durkin, M. S.; Dietz, P. M., Prevalence of Autism Spectrum Disorder Among Children Aged 8 Years - Autism and Developmental Disabilities Monitoring Network, 11 Sites, United States, 2016. *MMWR Surveill Summ* **2020,** 69, (4), 1-12.
3. Salari, N.; Rasoulpoor, S.; Rasoulpoor, S.; Shohaimi, S.; Jafarpour, S.; Abdoli, N.; Khaledi-Paveh, B.; Mohammadi, M., The global prevalence of autism spectrum disorder: a comprehensive systematic review and meta-analysis. *Ital J Pediatr* **2022,** 48, (1), 112.
4. Lotter, V., Epidemiology of autistic conditions in young children. *Social psychiatry* **1966,** 1, (3), 124-135.

5. Zuvekas, S. H.; Grosse, S. D.; Lavelle, T. A.; Maenner, M. J.; Dietz, P.; Ji, X., Healthcare Costs of Pediatric Autism Spectrum Disorder in the United States, 2003-2015. *J Autism Dev Disord* **2021,** 51, (8), 2950-2958.
6. Liptak, G. S.; Stuart, T.; Auinger, P., Health care utilization and expenditures for children with autism: data from U.S. national samples. *J Autism Dev Disord* **2006,** 36, (7), 871-9.
7. Cakir, J.; Frye, R.; SJ, W., The lifetime social cost of autism: 1990–2029. *Research in Autism Spectrum Disorders* **2020,** 72, 101505.
